# Impact of Chemicals and Processing Treatments on Thermo-Mechanical Recycling of Polyester Textiles

**DOI:** 10.3390/molecules30132758

**Published:** 2025-06-26

**Authors:** Zara Standring, Lisa Macintyre, Gigi Jiang, David Bucknall, Valeria Arrighi

**Affiliations:** 1 Institute of Chemical Sciences, School of Engineering and Physical Sciences, Heriot-Watt University, Edinburgh EH14 4AS, UK; zs20@hw.ac.uk (Z.S.); david.bucknall@hw.ac.uk (D.B.); 2 School of Textiles and Design, Heriot-Watt University, Galashiels TD1 3HF, UK; zj20@hw.ac.uk

**Keywords:** polyester textile waste, mechanical recycling, sustainability, textile to textile recycling, fibre to fibre recycling, fiber to fiber recycling, circular fashion, contamination, degradation

## Abstract

The textile industry is among the world’s largest, producing an estimated 124 million tonnes of fibres in 2023, with more than half of these being made from virgin polyester. Less than 0.1% of polyester fibres are recycled into new textiles at the end of their lives. Mechanical, thermo-mechanical, and chemical textile-to-textile polyester recycling are all technically possible, but thermo-mechanical recycling is reported to provide the most promising compromise between cost and quality. Myriad chemicals are used in polyester production, and this paper is the first to review the related academic literature to better understand their impact on recyclability. It has been demonstrated that chemicals used during the production and processing of polyester textiles can either provide resistance to, or catalyse, the degradation of polyester during thermo-mechanical recycling processes. However, the effect of combinations of these chemicals on recycling is largely unknown. Limiting, standardising, and transparently reporting the chemicals used during textile production would simplify research and could lead to better quality products after recycling.

## 1. Introduction

**Figure 1:**
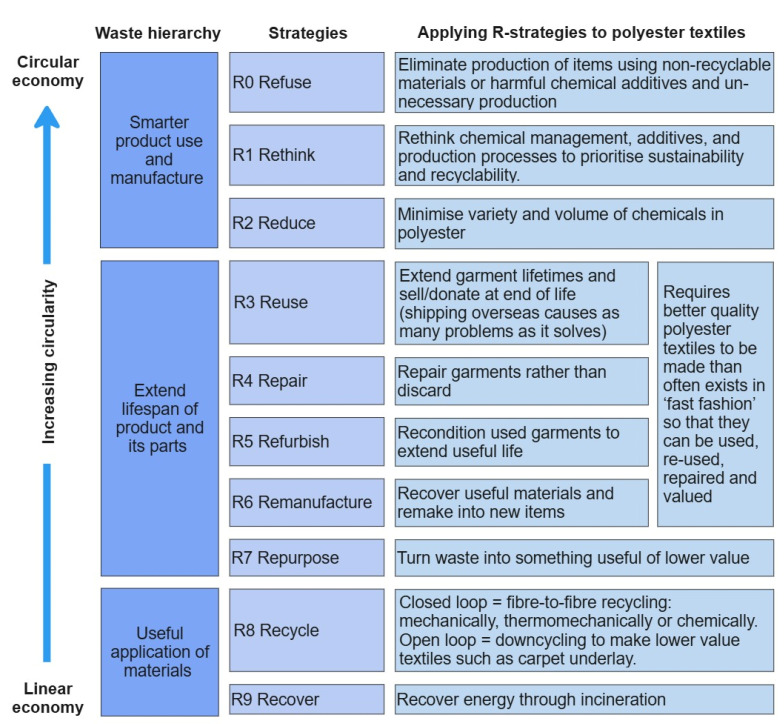
Optimising sustainability and circularity of polyester textiles, adapted from the R-strategies framework in reference [9].

The urgent need to make the textile industry more sustainable is now widely recognised [1,2,3,4,5,6,7]. Polyesters contribute to approximately 51–64% [1,2,3,4,5,8] of fibres produced globally, so increasing the sustainability of their production and developing strategies with high circularity (Figure 1, adapted from [9])) is of great importance to reduce the use of natural resources, waste, and environmental impact. Low-cost polyester textiles, however, are often of poor quality and are not suitable for the extended lifespan stages (R3–R7) as shown in Figure 1. This review focusses, therefore, on the impact of manufacturing choices (R0–R2) and on recycling efficiency (R8).

Although textile-to-textile recycling is technically possible, and necessary to deal with mounting waste, there are many hurdles to overcome before it becomes a reality. Commercial and infrastructural hurdles include optimising, scaling up, and reducing costs associated with collection, sorting, (cleaning), drying, shredding, and pelletising polyester in preparation for being made into new filaments, yarns, fabrics, and products. One of the major technical problems we have identified (through experimentation) is understanding why the quality and yield of recycled material vary between different textile sources of polyester. The novelty and contribution of this review is in documenting potential contaminants to polyester recycling from a wide range of papers. Having identified potential contaminants, we call for experimental research to propose and test polyester compositions that minimise degradation during recycling without compromising the quality and longevity of the virgin material.

There are three main methods of recycling polyesters—–mechanical, thermo-mechanical, and chemical [10,11]. Mechanical recycling (shredding and garnetting) significantly shortens fibre lengths, which reduces the quality of recycled material, resulting in limited applications and recycling cycles possible [12,13]. Thermo-mechanical and chemical recycling have the potential to produce quality recycled poly (ethylene terephthalate) (rPET), and multiple re-cycles may be possible [12]. Post-consumer rPET from plastic bottles has been increasingly used in textiles, but this does not address the textile waste problem, currently estimated at 84–92 million tonnes collected annually [1,4,14], with 66–75% ending up in landfills or being incinerated. In 2025, in the UK, many councils send most of their general/residual waste (including textiles) for incineration (R9 in Figure 1) with increasingly small fractions going to landfills [5,15,16].

Chemical recycling converts polyester back to its component parts, but it is expensive and requires auxiliary chemicals [17]. Thermo-mechanical recycling is the easiest method to implement as it is comparatively cheap, does not require additional chemicals, and uses existing equipment and processes [18,19]. However, degradation (thermal, thermo-oxidative, and hydrolytic) occurs during the thermo-mechanical recycling process, reducing the quality of the polyesters [13,20,21]. Hydrolytic degradation, or hydrolysis, is particularly problematic at the high temperatures required for melt-spinning even if there is little residual water, especially if acids or bases are present, as these can catalyse the degradation process [22]. For this reason, in PET production, the moisture level is controlled to be less than 0.005 wt% (50 ppm) [22,23], to minimise the level of degradation.

Less than 0.1% of polyester textiles are recycled in a closed loop process into new textiles [20,24] because large-scale, textile-to-textile recycling does not yet exist. One way to potentially increase recycling is by design [13], making polyester manufacturing smarter (R0-R2) with a particular emphasis on changes necessary to facilitate large-scale recycling (R8). Up until now, design for recycling has focused on components of garments (fabric, zips, and thread [25]) and less attention has been paid to chemicals used throughout the production of textiles.

A myriad of different chemical compounds (estimated to be 15,000–18,000 [14,26]) can be used in large volumes (273–580 g per kilogram of textile [26,27]) during textile production to improve the processability or properties of the fabric, including colour, strength, comfort, and flame retardancy. These chemicals frequently become contaminants in the recycling process.

A comprehensive list of additives used specifically in polyester production does not exist and compiling one is challenging. Many chemicals used in textiles are going unreported as studies in the literature often focus on banned and restricted chemicals [28,29,30,31,32,33,34,35,36,37,38], which constitute a minor proportion of those currently in use. Although, in the EU, the use of chemicals in textile products is regulated under REACH (Registration, Evaluation, Authorization and Restrictions of Chemicals), due to the complexity and global nature of the textile value chain, many ‘banned’ chemicals are still detected in garments [28].

The presence of additives and contaminants in textile waste adds complexity to the recycling process [10,13,39]. In recent reviews, the effect of additives on chemical recycling has been discussed [39,40], but there is little information on how contaminants in textile waste may affect other recycling processes. For thermo-mechanical recycling, some volatile chemicals will be removed during degassing and solid particles can be separated by filtration [41] (which reduces the proportion of material recycled). Although most additives are weakly bonded to PET fibres, others form covalent bonds and will interfere with any recycling process. Therefore, the effect of different contaminants on textile-to-textile recycling and the quality of recycled polyester needs to be evaluated. In this work, following a review of the literature, we identify potential chemical compounds, and classes used specifically in the production of polyester textiles and highlight the scale of the contamination problem.

## 2. Methodology

Given the magnitude of the problem in terms of number and variety of additives/contaminants that might be present in polyester textiles [42], we will not present a systematic or comprehensive review. The aim of the present work is to provide an overview of the possible implications associated with the presence of additives and contaminants on thermo-mechanical recycling. The review will:

- Identify potential risks with recycling contaminated PET textile waste during each stage of the manufacturing process;
- List and categorise the most common additives and contaminants present during the whole life cycle of a textile product;
- Give examples of the effects that various classes of contaminants may have on recycling;
- Present less chemically intensive alternative textile production methods with a critical analysis of their advantages and limitations where possible.

The data collection process involved searching both scientific (Web of Science, Science Direct) and grey (commercial and industrial reports) literature as well as textbooks. The keywords used, either individually or in combinations, were as follows: additives, contaminants, contamination, textiles, polyester, PET, recycling, finishing chemicals, finishing treatments, contamination, dyes, and dyeing. Most textile-to-textile recycling methods are currently in the research phase and are not yet commonly used in industry.

## 3. Additives in Textiles

Contamination can occur in every step of textile production, starting with the polymer synthesis. Contaminants can be intentionally added substances (IASs), such as catalysts, stabilizers, and residual monomers, or non-intentionally added substances (NIASs), i.e., impurities and by-products of polymerisation or degradation [43,44].

### 3.1. Polyester Structures

Polyester fibres are manufactured fibres in which the long-chain synthetic polymers are made up of at least 85%, by weight, ester of a substituted aromatic carboxylic acid, usually terephthalate units, and a diol [45,46]. Chemically, polyesters are a whole class of homo- and co-polymers that are produced by reaction between a diacid, e.g., terephthalic acid (TPA), and a diol, e.g., ethylene glycol (EG), to form an ester linkage [47]:
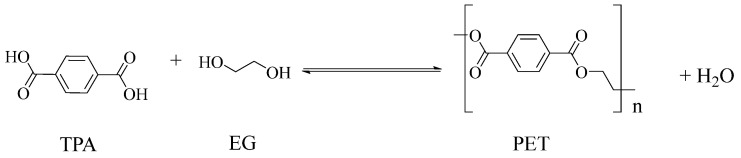


Table 1 lists the range of polyesters commonly used in the textile and manufacturing industries. Roughly 95% of polyester fibres produced worldwide consist of poly (ethylene terephthalate) (PET) [45]. Other polyesters, particularly poly (butylene terephthalate) (PBT) and poly (trimethlyene terephthalate) (PTT), are also used as fibres [46]. Differences in chemical structures (Table 1) affect thermal, mechanical, and other properties such as filament-forming ability, degree of orientation, and elasticity of the fibres [46]. For example, the presence of a naphthalate ring in poly(ethylene naphthalate) (PEN) increases the polymer’s rigidity and melting temperature, making it suitable for use in high-performance fibres [48]. One advantage of PEN compared to PET is the increased resistance to hydrolytic degradation. Thus, PEN is less prone to the molar mass decrease caused by degradation and deterioration of mechanical properties during extrusion. This makes PEN preferable to PET in terms of recycling but, being more expensive, its ability to compete with PET on a commercial scale is limited [49].

Co-polyesters (Table 2), synthesised from a mixture of monomers, provide enhanced properties over homopolyesters, e.g., in dyeability and flexibility. For example, cationic dyeable PET (CDP) is being increasingly used in industry as it offers an environmentally benign alternative to PET [50,51]. This is due to the more sustainable dyeing process, with water and energy savings. The presence of SIPE (Table 2) in the co-polymer structure of CDP introduces disorder, causing a decrease in thermal and hydrolytic stability of the co-polymer compared to PET [52]. The effect of this reduced thermal and chemical stability on recycling needs to be carefully evaluated.

PET flexibility can be increased by replacing part of the EG monomer with diethylene glycol (DEG) [53,54]. However, DEG units are more susceptible to thermo-oxidative degradation than EG units, causing scission and discolouration of the polymer chain [55]. Therefore, a greater ratio of DEG to EG units in a co-polyester would likely produce inferior recycled material. PET homopolymers contain 1 to 1.5 mol % DEG [21]. To improve recyclability of the polymer, DEG content needs to be kept as low as possible although this may compromise flexibility and potentially the types of applications.

**Table 1:** Chemical structures of common homo-polyesters with their monomers. Properties are shown in relation to PET and structures [56].

Polyester *	Diacid(s)	Diol(s) ^(b)^	Selected Properties
Poly(ethylene terephthalate) (PET) 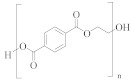	TPA or DMT ^(a)^	EG	---
Poly(butylene terephthalate) (PBT) 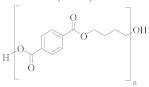	TPA or DMT	BD	Greater alkaline hydrolysis resistance, crystallises faster [57]
Poly(trimethlyene terephthalate) (PTT) 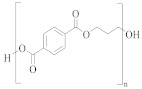	TPA or DMT	PD	Greater alkaline hydrolysis resistance, crystallises faster [57]
Poly(cyclohexanedimethylene terephthalate) (PCT) 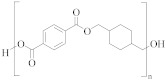	TPA or DMT	CHDM	Processing temperatures > 300 °C, near decomposition temperature [58]
Poly(ethylene naphthalate) (PEN) 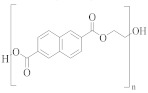	naphthalene-2,6-dicarboxylic acid	EG	Greater alkaline hydrolysis resistance
Poly(ethylene 2,5-furandicarboxylate) (PEF) 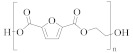	TPA or DMT	EG	Lower melting temperature, derived from biomass [59]

* Some of the polyesters are more commonly used as thermoplastics rather than in textiles [60]. They are listed here as non-textile polyester components are used in garments and may be relevant to the recycling process. ^(a)^: TPA = terephthalic acid and DMT = dimethyl terephthalate. ^(b)^: EG = ethylene glycol, BD = butane-1,4-diol, PD = propane-1,3-diol, and CHDM = 1,4-cyclohexanedimethanol.

**Table 2:** Chemical structures of common co-polyesters with their monomers. Selected properties are listed to highlight differences in relation to PET [58].

Polyester	Diacid(s) ^(a)^	Diol(s)	Selected Properties
Cationic dyeable PET (CDP) 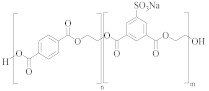	SIPE and TPA	EG	Lower resistance to thermo-oxidative degradation [61]
poly(ethylene terephthalate-co-diethylene terephthalate) 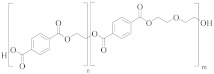	TPA or DMT	EG and DEG (1–4 mol%)	Lower resistance to thermo-oxidative degradation [53,54]
poly(ethylene terephthalate-co-isophthalate) (CPET) 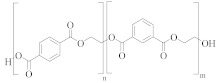	isophthalic acid (IPA) (<5 mol%) and TPA or DMT	EG	
poly(ethylene-co-1,3-butylene terephthalate) 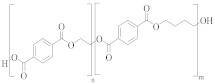	TPA or DMT	EG and butane-1,4-diol (<5 mol%)	Greater alkaline hydrolysis resistance, crystallises faster [57]
poly(ethylene-co-1,3-propylene terephthalate) 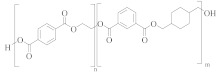	TPA or DMT	EG and propane-1,3-diol (<5 mol%)	Greater alkaline hydrolysis resistance, crystallises faster [57]
poly(ethylene-co-1,4-cyclohexanedimethanol terephthalate) (PETG) 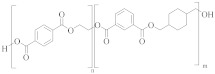	TPA or DMT	EG and CHDM (≤50 mol%)	Greater hydrolysis resistance [62]
Poly(cyclohexanedimethlyene terephthalate-co-isophthalate) (PCTA) 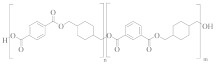	TPA and IPA (>35 mol%)	CHDM	Greater hydrolysis resistance [58]

^(a)^: SIPE = 5-bis(hydroxyethyl)-isophthalate, TPA = terephthalic acid, DMT = dimethyl terephthalate, and IPA = isophthalic acid.

### 3.2. Contaminants from Synthesis and Polymer Processing

PET is produced by a two-step polycondensation process between EG and TPA or DMT, although the former is predominantly used [56]. In this case, the direct esterification of TPA and EG gives a pre-condensate, mainly bis(2-hydroxyethyl) terephthalate (BHET) with some oligomers. A polycondensation reaction is then carried out under vacuum, at 230 to 260 °C, in the presence of an intentionally added catalyst [46,56]. Due to these high temperatures, monomer degradation and side reactions can occur [63]. Oligomers of less than 1,000 g/mol are formed as side products during polymerisation [64], including cyclic trimers that can negatively impact the dyeing and spinning of filaments [60].

**Figure 2:**
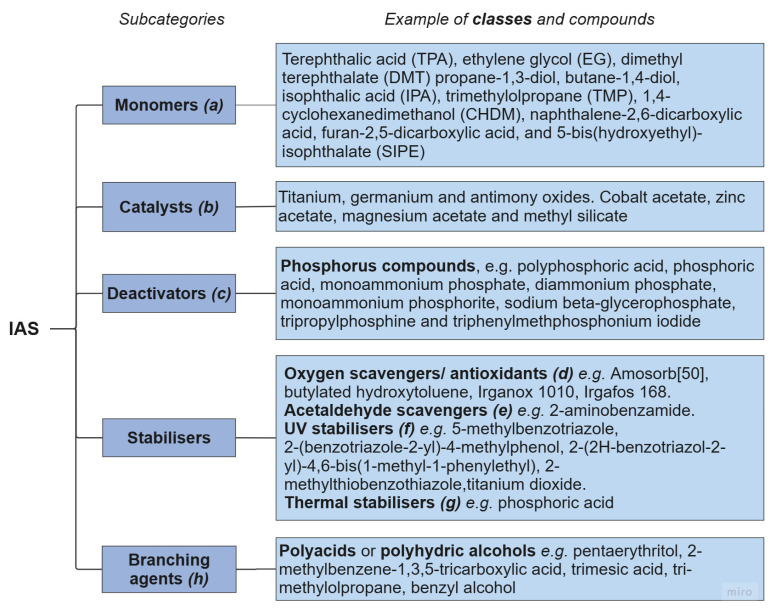
Major categories of intentionally added substances (IASs) in polyester textiles. Examples of chemical compounds for each category are given. References: ***(a)*** [65]; ***(b)*** [63,66]; ***(c)*** [66,67]; ***(d)*** [68,69,70]; ***(e)*** [69]; ***(f)*** [28,34,71]; ***(g)*** [21]; ***(h)*** [72,73].

Chemicals used during the polymerisation process, i.e., monomers, catalysts, and stabilizers (Figure 2), are classed as intentionally added substances (IASs). These, if present in the final product, become contaminants in recycling. For example, small amounts of monomers and/or oligomers may be present in the polymeric product due to an incomplete polycondensation reaction of EG and TPA or DMT [65].

Figure 2 lists the major categories of IAS found in PET textiles, some of which are discussed below.

Antimony trioxide, a common catalyst in PET production, is present in 80–85% of all virgin PET [74]. Biver et al. [74] reported total concentrations of antimony in polyester fabrics ranging from 125 to 470 μg g^−1^.

Titanium dioxide (TiO_2_) is also used as a catalyst and may be added during the extrusion process to make fibres opaque and absorb UV-B rays [46,60,75]. The effect of the catalyst on the discoloration of PET was studied by Yang et al. [76] using a range of spectroscopic techniques. These authors reported that TiO_2_ caused greater discolouration compared to antinomy-containing PET due to the production of quinoid species during thermo-oxidative degradation. This is not expected to have adverse effects on textile recycling as dyeing could mitigate the impact of discolouration.

Generally, the catalysts used can affect the types of side reactions and by-products as shown by an Extended X-ray Absorption Fine Structure or EXAFS study carried out by Nishioji et al. [77].

Plasticizers are rarely used in PET-based textiles [78]. However, di(2-ethylhexyl) phthalate (DEHP), a plasticizer commonly associated with poly(vinyl chloride) (PVC), has been detected in textiles, including PET fibres, at a level of 2–8 mg/kg [79]. One study by Saini et al. [80] reported a total phthalate concentration in polyester fabric of 1950 ng/dm^2^, with 1091 ng/dm^2^ attributed to DEHP. There have been multiple studies on health risks associated with exposure to DEHP and other phthalates, including work on their presence in recycled plastic [81], but no information regarding their effect on the properties of recycled polyester was found.

Heavy metal catalysts are used in industry for polyester synthesis [46,56] despite concerns over their toxicity [44]. Deactivators, often phosphorus compounds, may be added between the two polymerisation steps to deactivate the first catalyst [67] or before polymer processing to reduce discolouration and degradation [66]. Phosphoric acid, used as a deactivator and thermal stabilizer, has been reported to increase the tensile strength of polyester yarn by 15% and elongation by 88% [52]. Residues of the catalysts and deactivator may affect properties of the final product such as dyeability and transparency [77].

**Figure 3:**
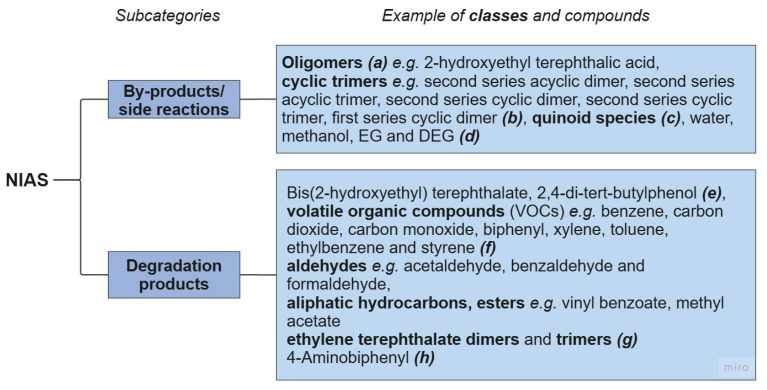
Major categories of non-intentionally added substances (NIASs) in polyester textiles. Examples of chemical compounds for each category are given. References: ***(a)*** [64]; ***(b)*** [60,69]; ***(c)*** [76]; ***(d)*** [82]; ***(e)*** [64]; ***(f)*** [21,83]; ***(g)*** [83]; ***(h)*** [69].

NIASs are produced through the degradation of a polymer or side reactions during synthesis. Therefore, different polyesters are expected to generate different types and different quantities of NIASs (Figure 3). For example, DEG is produced in a side reaction during PET synthesis [82]. The sulphonate groups in the SIPE monomer, used to synthesise cationic dyeable PET [61], can catalyse this side reaction, increasing the production of DEG [21,82]. A higher content of DEG can lead to greater degradation. Therefore, co-polyesters containing SIPE or large amounts of DEG could produce an inferior recycled product compared to pure PET. As noted earlier, an in-depth assessment of the recyclability of cationic dyeable PET is needed.

### 3.3. Recycled PET (rPET) in Textiles

Currently, 12.5–15% [24,84] of polyester fibres are made from recycled materials, ca. 98% of which come from recycled plastic bottles [10,24]. There are several reasons why rPET from bottles is used as a feedstock for new textiles [85]. First of all, it is relatively pure compared to textile and other plastic waste. The similarity between rPET from bottles and virgin PET means that simple adjustments are needed in switching to rPET feedstock. A further important factor is that the molar mass of textile-grade PET is in the range of 15,000 to 27,000 g mol^−1^, much lower compared to that of thermoplastic PET used in plastic bottles and packaging [46,85,86]. For this reason, recycled PET from plastic bottles, despite the occurrence of degradation during recycling, meets requirements for use in new textiles.

**Figure 4:**
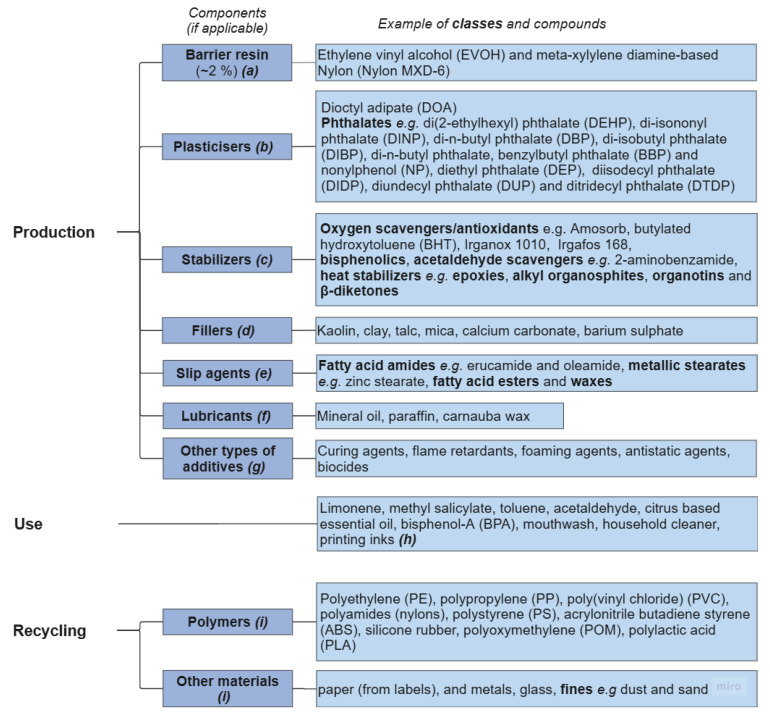
PET plastic contaminants from production, use, and recycling. References: ***(a)*** [68]; ***(b)*** [43,79,81,89,90]; ***(c)*** [68,69,70,83,90]; ***(d)*** [69]; ***(e)*** [83]; ***(f)*** [43]; *(g)* [69]; ***(h)* [44,91]**; ***(i)*** [68,92].

Considerable research has been carried out on PET bottle recycling [87]. Problems associated with the many chemical substances, i.e., the additives, present in plastics have been highlighted (Figure 4). These range from potential soil, water, or food contamination to risks of human exposure, release during recycling, and their effects on recyclates [88]. A list of common plastic additives is given in Table 3.

Roughly 600 additives have been authorised for use in plastics that come into contact with food and several compounds used in food production, including limonene (citrus-based essential oil) and methyl salicylate (oil of wintergreen), have been found in rPET [44].

One class of additives, common in packaging but rarely used in textiles, includes plasticizers. Common plasticizers such as di-n-butyl phthalate (DBP) [43] have been detected in PET plastic bottles [93]. Depending on the storage conditions, DBP can leach out, potentially causing serious health risks [90,94]. However, according to the PET Resin Association (PETRA) [95], phthalates are not used in the manufacturing of PET bottles and should not pose any issue in the recycling process.

A possible rPET contaminant is bisphenol-A (BPA) from printing inks, mouthwash, and household cleaners [44]. One study by Dreolin et al. [91] reported up to 10 ppm BPA in rPET. BPA is not directly used in textile products but can be an intermediate in dye and antioxidant production. BPA derivatives have also been used as finishing agents. Xue et al. [32] detected BPA in their study on textiles and infant clothing. Its presence was believed to be due to the use of rPET from plastic bottles in garments such as socks, which contained 13 ppm of BPA [32].

rPET often contains trace amounts of other polymers and impurities [92] (Table 3), a result of sorting and separation methods not being 100% efficient, e.g. sorting efficiency using the float method is reported to be 95–99 wt% [96]. Thoden van Velzen et al. [68] reported rPET to contain polyolefins (PE/PP), PVC, and polystyrene (PS) in addition to paper (from labels), dust, and metals.

Contamination with polypropylene (PP) and PVC can have a negative impact on the properties of the recyclates, e.g., a loss of transparency in the final product [97]. For example, PVC degrades at high temperatures, releasing hydrochloric acid, which catalyses the hydrolysis of PET, causing a decrease in molar mass during recycling [98]. The presence of PVC, PP, and polyethylene (PE) in rPET can lead to fluctuations in the pressure during extrusion [68], adding difficulty to processing. Additional information on the effect of polymer impurities on rPET is given in Table 3.

Despite the significance of IASs and NIASs in polyester textile-to-textile recycling, this is still a largely unexplored area and a detailed understanding is lacking.

### 3.4. Processing Polyester Yarns for Fabric Production

**Figure 5:**
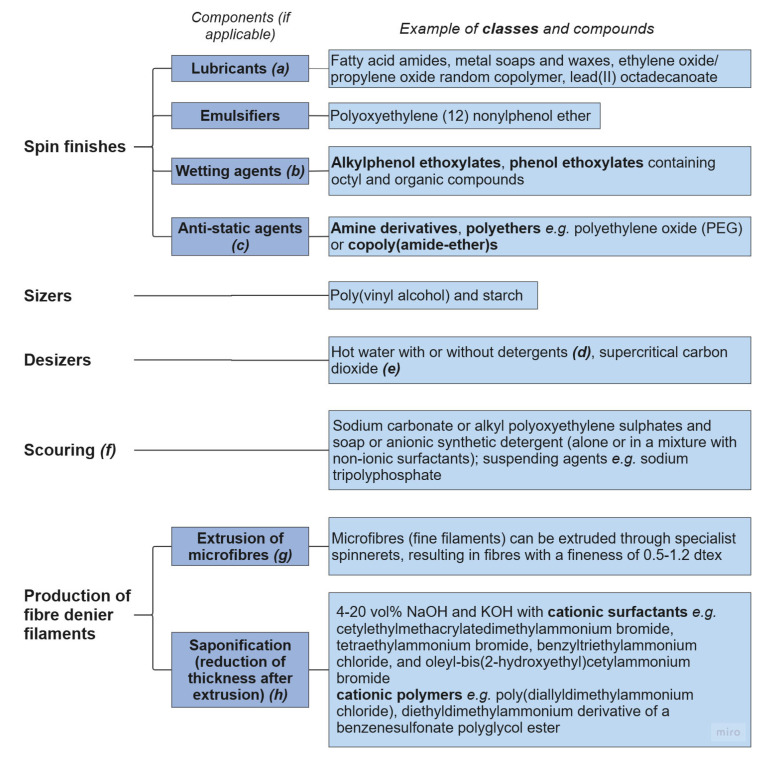
Contamination from colouration of polyester textiles. References: ***(a)*** [43]; ***(b)*** [101]; ***(c)*** [102]; ***(d)*** [27]; ***(e)*** [103]; ***(f)*** [104]; ***(g)*** [105]; ***(h)*** [106].

Various chemicals are added to polyester filaments/yarns to aid the production of the final fabric [99]. Although many of these additives are subsequently removed, removal methods are not completely effective, so residues will remain in the final product, contaminating the textile and ultimately the recyclate [100]. Examples of the chemicals used in all processing treatments are presented in Figure 5.

#### 3.4.1. Spin Finishes, Sizing, and Pretreatment

Two common treatments used to improve the processability of polyesters are spin finishes and sizers, with examples shown in Figure 5. Spin finishes, applied to filaments before drawing, consist of lubricants [43], emulsifiers, wetting agents, and anti-static agents [60,107]. Alkylphenol ethoxylates, phenol ethoxylates containing octyl and organic compounds, are used as wetting agents [101]. Typical industrial lubricants include fatty acid amides, metal soaps, and waxes [43].

Sizers provide abrasion resistance to filaments/yarns for weaving [27,108]. Spin finishes and sizers affect the dyeing process, so they are commonly removed by scouring [60,108], a cleaning process that removes impurities [14,104] after production. Scouring can be performed with sodium carbonate (Na_2_CO_3_) or alkyl polyoxyethylene sulphates [104]. As sulphonate groups catalyse DEG production, scouring with Na_2_CO_3_ instead of polyoxyethylene sulphates could improve the recyclability of PET-based textiles.

The choice of chemicals for the processing treatments can ultimately affect the quality of the recycled material. Residues of spin finishes have been extracted from commercial PET fabrics [109]. Rahman et al. [110] found that the rate of PET hydrolysis decreased from 6.19%/h to 4.2%/h due to spin finishes. Their presence increased PET’s resistance to alkaline hydrolysis by preventing the penetration of sodium hydroxide (NaOH). Attempts were made to identify the spin finish components. Unfortunately, they were unsuccessful.

#### 3.4.2. Chemical Reduction of Linear Density (Optional Process)

Reducing the linear density (denier or dTex) of polyester fibres improves the feel and performance of the textile. Filaments less than 1.2dTex are known as ‘microfibres’, or microdenier fibres, and are common in better quality apparel and other textiles. Fine filaments can be achieved either by extruding microfibres, which is more expensive but requires no auxiliary chemicals, or by reducing the denier of standard filaments with saponification. Alkaline hydrolysis or local saponification can be used to modify the surface of PET yarns and reduce thickness. The saponification of PET increases the number of carboxyl end groups at the surface allowing the movement of water [60,111], giving improved wicking properties. Phaneuf et al. [112] reported that 0.5–1 vol% sodium hydroxide (NaOH) produced carboxyl end groups at the surface without reducing the mechanical properties of the fibres. However, saponification is usually performed with 4–20 vol% NaOH, causing the unwanted degradation of the core PET fibres [106]. The presence of cationic surfactants can also accelerate the production of carboxyl end groups [113]. These carboxylic end groups will catalyse degradation during recycling [21,114].

Extra-fine polyester fibres can also be achieved by extruding microfibres (0.5–1.2 dtex [105]). This is a chemical-free alternative that is relatively expensive but likely to have no adverse effects on recycling. Regardless of the production method, microfibres have an increased surface-area-to-volume ratio, requiring more dye to achieve the same shade of colour compared to thicker fibres [115]. Dyeing is one of the most chemically intensive processes in the textile industry [116]. Research is needed to improve the dyeing process of microdenier PET fibres and develop technologies that reduce the environmental impact, e.g., dope dyeing or using supercritical CO_2_ [117].

#### 3.4.3. Heat Setting for Textured Multi-Filaments

Polyester filaments can be extruded as monofilaments or multi-filaments. Most multi-filaments are textured before use to improve handle, functionality, and processibility. Texturising involves crimping, or other means of adding texture to, the filaments while they are heated to temperatures above the onset of hydrolysis (~100 °C [21]) so that degradation may occur. Thermal treatments can also lead to the diffusion and evaporation of some low-molecular-weight (<300 g mol^−1^ [109]) contaminants [110].

### 3.5. Colouration of Polyester Yarns and Fabric

There are approximately 100,000 commercial dyes [101]. This is an area affected by many changes: new dyes are continuously being developed or modified; others have been banned due to health risks but are still being detected in some textiles [42].

**Figure 6:**
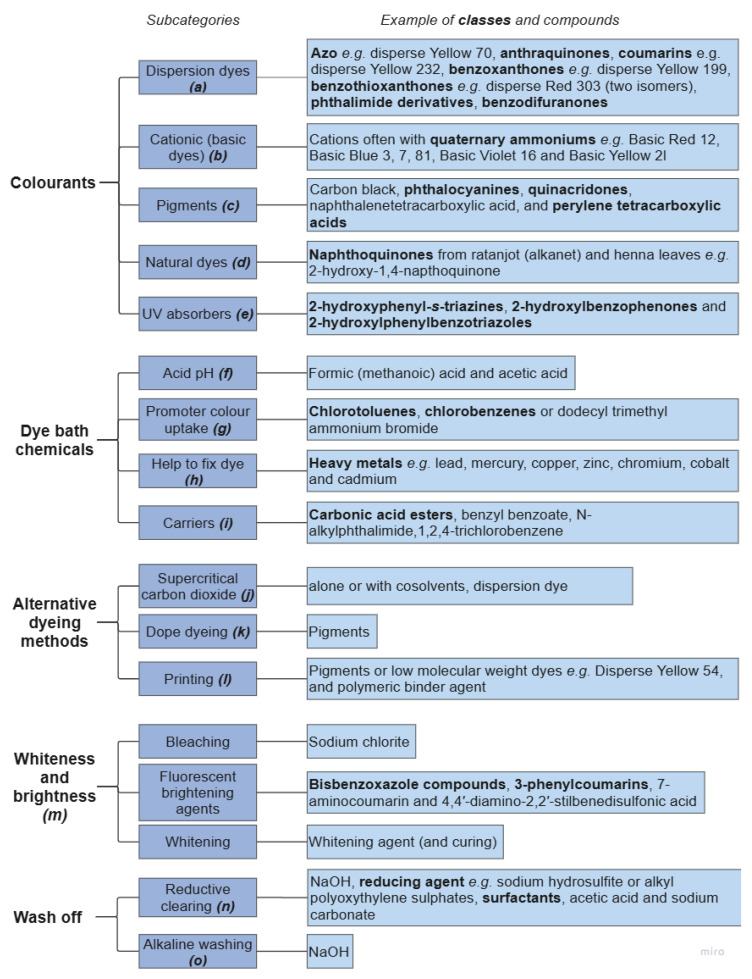
Contamination from colouration of polyester textiles. References: ***(a)*** [126,127]; ***(b)*** [119]; ***(c)*** [128]; ***(d)*** [129]; ***(e)*** [130]; ***(f)*** [131,132]; ***(g)*** [131]; ***(h)*** [14]; ***(i)*** [27]; ***(j)*** [133]; ***(k)*** [124]; ***(l)*** [126,131]; ***(m)*** [104]; ***(n)*** [131,134]; ***(o)*** [126].

Polyesters can be coloured using different dye classes [118]: disperse [119], cationic [120], natural [121,122,123], or pigment [124] (Figure 6). Natural dyes are often considered more environmentally friendly, can be biodegradable, and can have greater compatibility with the environment [123]. The chemical structure of the dyes dictates their thermal stability and the by-products that could be released. For example, azo dyes can degrade to produce aniline [101], amino acids [101], and amines [36]. Natural dyes are particularly susceptible to light and elevated temperatures. For instance, Arora et al. [121] reported that almost 90% of a natural dye, extracted from Ratanjot root, decomposed at 130 °C. Although this dye showed good wash and rub fastness, it was also sensitive to light. Therefore, natural dyes may not offer a suitable alternative to synthetic ones for polyester. Pigments, e.g., phthalocyanines, have higher thermal stability (decomposition temperature > 400 °C [125]) than dyes, so they are unlikely to degrade during extrusion, which may be advantageous for thermo-mechanical recycling.

Polyester textiles can be dyed using different methods although the most common and chemically intensive is using dye baths. The harsh conditions of dye baths can fully or partially remove contaminants incorporated into the fibres in earlier steps. For example, roughly 30% of the antimony oxide catalyst present in PET is believed to be removed during dyeing at 130 °C [27,74]. Alternative dyeing methods, like dope dyeing and supercritical CO_2_ (ScCO_2_) reduce the number of auxiliary chemicals needed and therefore decrease potential contamination [26]. ScCO_2_ dyeing is carried out at a lower temperature compared to traditional dyeing processes, reducing energy consumption. However, there are cost and infrastructure limitations. For example, ScCO_2_ dyeing requires initial investments to purchase specialised equipment. Dope dyeing uses no water, is cheaper and less energy-intensive than conventional bath dyeing, but choosing the right pigment is crucial to avoid the deterioration of mechanical properties. The addition of pigments during dope dyeing to the polymer matrix has also been reported to reduce the strength of the fibres by 2–5% [124]. But dyes and dye methods are not commonly reported to fashion brands, let alone consumers or downstream processors, so their effect on recycling is difficult to determine.

Wash-off is required to remove residual dye from the surface of the fibre after bath and ScCO_2_ dyeing [131] and can be achieved either with reduction clearing or a milder alkali wash [126]. Dyes that are hydrolysed in weak alkaline solutions are considered more environment friendly [126]. The advantages of reduction clearing include the removal of oligomers and other contaminants [134,135]; however, the process is also likely to cause alkaline hydrolysis, which could reduce the quality of the PET (e.g., molar mass and intrinsic viscosity). Recent work has reported oligomer removal rates using ozone treatment equal to those of reductive clearing whilst being more energy- and water-efficient [136]. Enzyme-based reductive clearing processes are also effective at removing residue dyes and oligomers, but scalability may be limited by the high cost of enzymes [137].

### 3.6. Fabric Finishing Agents

**Figure 7:**
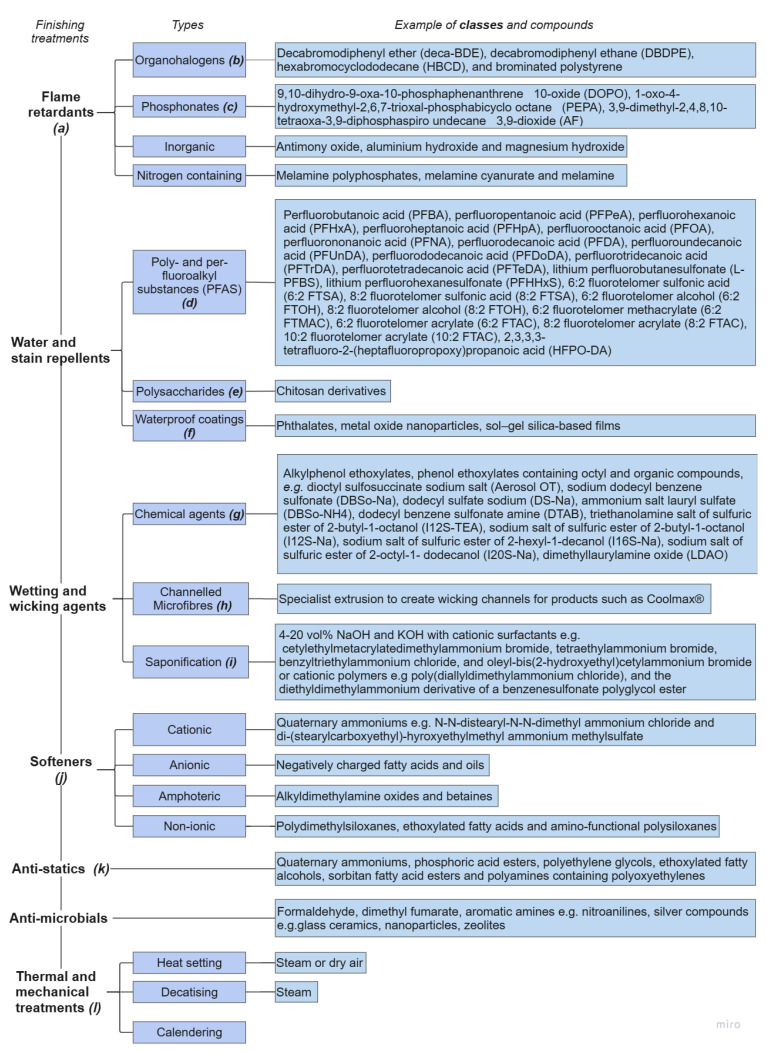
Contamination from finishing treatments of polyester textiles. References: ***(a)*** (1): [142]; ***(b)* [139,142]**; ***(c)*** [71,140]; ***(d)*** [30]; ***(e)*** [143]; ***(f)*** [144,145]; ***(g)*** [101,107]; ***(h)*** [146]; ***(i)*** [106]; ***(j)*** [71,108]; ***(k)*** [147]; ***(l)*** [148,149].

Finishing agents are often added to textiles once they have been woven or knitted into a fabric to impart additional properties. An estimated 3,000+ finishing agents are used in the textile industry [108], with the most common ones being listed in Figure 7. These should be considered as potential contaminants with largely unknown impacts on recycling.

Finishing agents can be classed according to the properties they impart to the fibre, e.g., flame retardants or anti-microbials [138]. They can be absorbed into the fibre or adhered to by reaction [108].

#### 3.6.1. Flame Retardants

The two most common classes of flame retardants are organohalogens [139] and phosphonates [60] (see Figure 7). Halogenated (organohalogens) flame retardants, particularly those containing bromine, have been restricted or banned [43] for health and environmental reasons. An alternative to halogenated compounds includes phosphorus-based flame retardants (P-FR). Bascucci et al. [140] found that PET containing one type of P-FR decomposed at high temperatures accelerating degradation compared to pure PET. PET containing a different P-FR showed evidence of branching and chain extension as indicated by changes in molar mass compared to pure PET (a decrease in one case, and an increase when using the other P-FR). Similarly, Chen et al. [141] investigated the recyclability of PET containing two phosphorus flame retardants. They reported very different effects on PET recyclability with one of these flame retardants causing excessive chain branching that prevented rPET being melt-spun into filaments. By comparison, the other retardant offered good recyclability, indicating that the choice of flame retardant could have a large impact on the recycling PET textiles.

#### 3.6.2. Wetting/Wicking

Enhancing the wetting and wicking properties of polyester garments improves the comfort and moisture flow in clothing [60,111]. This is particularly important for certain textiles, e.g., sportswear, to remove perspiration and reduce odour and bacterial buildup [60,148].

Wetting refers to the spreading of a liquid on the surface of a fabric, and it is controlled by the liquid surface tension, the surface energy of the fabric, and other parameters [150]. Wetting agents act by reducing the surface tension of liquids, allowing for greater absorption into the fibres [107].

Polyester is hydrophobic and has no inherent wetting or wicking ability. It can be extruded through specially shaped spinnerets to enhance wicking. This can also be achieved by extruding thinner fibres (0.5–1.2 dtex [105]) that have better moisture transmission properties [150]. Alternatively, standard fibres can be made thinner using saponification (see Section 3.4.2).

More recently, modifications to the fibre surface have been made using ozone/UV or plasma treatments [151]. These treatments increase the number of hydroxyl and carboxyl groups at the surface, which increases wettability [152], whilst reducing the environmental impact by decreasing chemical usage [123]. However, these treatments like saponification increase the number of carboxyl groups, leading to greater degradation during recycling.

Wetting and wicking agents (examples shown in Figure 7) can be combined into a single finishing treatment in a spin finish, during dyeing, sprayed on the final fabric or added in a pad–dry–cure finishing process. If enhanced wicking properties are necessary, specially extruded PET microfibres as opposed to a finishing treatment may prove advantageous to reduce the contamination of PET textiles. As discussed in Section 3.4.1, any alkaline treatment could negatively impact recycling, but there has been a lack of studies in this area.

#### 3.6.3. Stain- and Water-Repellent Additives

Poly- and per-fluoroalkyl substances (PFASs) are the most common water and stain repellents [14,153], with examples shown in Figure 7. They are a class of over 12,000 compounds [154], each containing strong carbon–fluorine bonds that do not readily degrade and are often incorporated into outdoor clothing and uniforms [154]. Water repellents for textiles are commonly side-chain-fluorinated polymers (SCFPs) with urethane or acrylate backbones [155].

The degradation of side-chain-fluorinated polymers can lead to the formation of non-polymeric PFAS [156], adding to the volume of fluorinated contamination that can be found in textiles. A study by van der Veen et al. [30] identified 22 PFASs in three different PET textile samples.

Alkaline hydrolysis can be used to remove some volatile PFASs such as fluorotelomer alcohols (FTOHs), but the treatment is not effective in removing side-chain-fluorinated polymers [154].

Like with halogenated flame retardants, there are serious concerns over the safety of PFAS compounds [157,158,159,160]. The European Chemicals Agency (ECHA) has released a report proposing a ban of all PFASs (over 12,000) [158]. This is stimulating work to find alternatives, e.g., polysaccharides [143], although previous studies conducted by the Danish Environmental Protection Agency were unable to identify suitable options that matched PFASs’ performance on all parameters [158].

#### 3.6.4. Thermal and Mechanical Finishes for Polyester Fabric

Polyester fabrics frequently undergo thermal and mechanical finishing treatments such as heat setting. This is performed to attain dimensional stability, and treatments can also imbue shape, e.g., pleats. For example, heat setting is often performed at a temperature in the range of 130 to 140 °C in steam or 190 to 220 °C in dry air under tension for a short period of time [148]. The implications of these elevated temperatures are described in Section 3.4.3 [21]).

### 3.7. Use Phase

The three main types of contamination during the use phase of PET-based textiles involve degradation, stains, and detergents.

Textiles undergo mechanical degradation due to wear and tear and chemical degradation during laundering and UV exposure [161]. UV stabilisers are often added to polyester garments to protect them against UV exposure [162] (Figure 2). Despite this, the photodegradation of polyester may still occur and exposure to UV radiation can increase the release of microfibres [163]. Other environmental factors can also cause the degradation of PET fabric components, e.g., bacteria degrade azo dyes, releasing amines [36].

Mechanical degradation will physically shorten the polyester fibres whereas chemical degradation may cause the scission of the polymer chain, releasing oligomers and small molecules (NIASs, Figure 3). Non-ionic surfactants in laundry detergents, e.g., polyethoxylated nonylphenols, can degrade forming toxic compounds such as nonylphenol (NP) and octylphenol (OP) [70]. Furthermore, additives and finishing agents can degrade during use, releasing various compounds that become NIASs [44,70].

Whilst polyester textiles are in use, they can absorb and release chemicals. PET textiles without stain repellent finishes are readily stained by contaminants including soil, deodorant, perfume, and oil [10,164]. Polyester fabrics can absorb low-molecular-weight chemicals they come into contact with [68], e.g., phthalates, brominated flame retardants, and organophosphate esters [80]. Chemicals can also migrate out of textiles, mediated by other factors such as sweat [74]. For example, up to 2% of the total antimony oxide catalyst in PET textiles was found to migrate into sweat [74,89], which can be washed away. Luongo et al. [165] showed that repeated laundering reduced the concentration of various contaminants including benzothiazoles and benzotriazoles. However, the textiles were kept in sealed bags between washes, which the prevented absorption of contaminants. This suggests that the level and type of contaminants may change throughout use.

### 3.8. Contamination During Recycling

Currently, less than 0.1% of textile fibres are recycled back into new textiles [20], so there is virtually no information on possible contamination arising from to textile-to-textile recycling. However, contamination from inaccurate sorting and separation is likely [166]. Currently, post-consumer textile waste is sorted manually [167,168,169] and is reliant on labels, which are frequently incorrect [170], missing, or imprecise. Garment labels can legally report 100% single-fibre content with 5% allowance for other fibres if ‘carded’ and more for trims or threads made from different fibres [171]. Sorting methods based on near-infrared (NIR) and visible spectroscopy are widely used to identify different types of plastic waste but suffer from various limitations. For example, Matoha Fabritell scanners have an accuracy of ± 10% for blends and ± 5% for pure fibres [172].

The contamination of PET with synthetic fibres during thermo-mechanical recycling due to inadequate sorting would likely lead to effects like those reported for rPET in Table 3. Further issues may arise from contamination by natural fibres, e.g., cellulose-based, which, due to their low decomposition temperature, could form black/burnt particles [68] during re-processing.

Contamination is not only an issue for thermo-mechanical recycling but also affects chemical recycling as the recovered monomers must be pure before they can be re-polymerised [40]. The purification process is considered safe and of sufficiently high efficiency that the FDA does not require the submission of cleaning efficiency data [173,174]. However, the purification process is time-, energy-, and cost-intensive and this is given as the main reason why chemical recycling is not currently economically viable [17]. Prior to chemical recycling, textiles are often washed to remove processing acids and finishing treatments to increase reaction rates and processability [10]. However, it has been demonstrated that chemicals used during processing can either provide resistance to [110] t or catalyse [106] the degradation of PET. In the case of chemical recycling, it may be advantageous to use chemicals that act as catalysts for PET degradation to potentially reduce the time and volume of further chemicals needed for full conversion into monomers.

Textile design and production should consider the entire life cycle and be tailored to optimising both the intended end-use and the end-of-life recycling process. For example, if thermo-mechanical recycling is the likely route, chemicals that catalyse degradation should be avoided.

## 4. Recycling Challenges

This paper has highlighted some of the important issues that are currently preventing large-scale textile recycling. Chemicals introduced during the different stages of textile manufacturing, and therefore potential contaminants of polyester recyclate, were identified. The current knowledge on the impact of additives and contaminants on PET textile recycling is very limited. Recent reviews have focused on the potential health and environmental risks of different additives used in the textile industry [88,175], but significantly more research is needed to understand their impact on recycling processes [2,99]. The effects of combinations of these contaminants on recycling are also largely unknown.

The textile industry has complex supply chains [1,6]. PET textiles would normally be processed by 3–6 independent suppliers before being delivered to the garment or product manufacturer. Therefore, most brands and garment manufacturers have no information on the chemicals and quantities used during textile production and cannot pass this on to potential recycling companies.

Radio frequency identification (RFID) threads can act as digital passports. They could potentially detail all the chemicals and treatments used during processing [176], and also be used to aid separation of textiles for recycling [177]. However, the RFID threads could themselves become contaminants, and, more importantly, in the absence of legislative requirement, it is unlikely that companies will adopt them, principally because of cost.

A more realistic method of increasing the recyclability of PET textiles in terms of contamination is by dilution. Virgin PET is frequently used to increase the viscosity of recycled PET, but it also mitigates the effects of certain contaminants [141,178]. However, reliance on dilution with virgin PET means that recycling is only partially ‘circular’ and significant volumes of waste polyester would still be incinerated or dumped.

## 5. Outlook

Polyester textiles are never pure as they can contain other polymers, IASs, and NIASs. The production of polyester textiles involves a vast number of chemicals that can become, react, or degrade into problematic contaminants for recyclers. As discussed in the review, some textile processes, e.g., scouring, dyeing, and wash-off, can partially remove potential contaminants. However, these processes are not completely efficient, and residues will remain. Some chemicals are also removed from the textile by migration during use, but additional contaminants may be absorbed from the environment, wearer, or laundry. Further, the recycling process has the potential to increase the number of contaminants due to polymer and/or additive degradation and insufficient sorting. Very little research on the effects of contaminants on thermo-mechanical recycling has been reported to date, making it difficult to set limits on the types or volume of contaminants tolerable for commercially viable recyclability. However, the following general recommendations are suggested based on the findings of this work:

1. Textile production relies on specific additives for processing and in finishing treatments to increase durability. Completely removing chemicals in textiles is not feasible. Instead, research needs to be conducted to understand which chemicals catalyse degradation and find alternatives.
2. Many of the less chemically intensive alternatives are more environmentally sustainable but currently have infrastructural issues and/or cost barriers to implementation.
3. Some additives can increase resistance to certain types of degradation. Identifying these chemicals and encouraging their use in textiles could potentially increase the quality of recycled products.
4. Polyester textiles should be designed to be recycled; chemicals and contaminants should be minimised and standardised wherever possible. Any chemicals used in PET production should be communicated to the brands, upstream manufacturers, and consumers in a permanent and readable manner.
5. A ‘plastic textiles’ treaty, like the plastics treaty currently in negotiation, could benefit recyclers and the environment.
6. PET specific recommendations:
7. Limit DEG content in the original polymer as it significantly reduces the molecular weight of PET during reprocessing.
8. Minimise PVC contamination. The hydrochloric acid formed during degradation can catalyse the hydrolysis of PET, reducing molecular weight.
9. Avoid any concentrated alkaline treatment. The resulting increase in the number of carboxylic end groups is known to catalyse the hydrolysis of PET.
10. Wicking and wetting agents should be avoided in favour of specially extruded (rather than alkaline-treated) channelled microfibre polyester, which exhibits similar properties.

Significantly more research is needed on the sources, types, and impacts of polyester contaminants for recycling.

When designing materials for recycling (as well as first-life performance), there are three major considerations: technology, education, and legislation. Firstly, the textile industry has developed as an intrinsically linear model, and both the chemicals currently in use and processes need to be reassessed to move towards a circular economy [179]. If we were to start designing processes for clothes and other textiles with recyclability in mind today, we would certainly make different choices. Nevertheless, there is great potential to reduce the environmental impact during production, i.e., the first cycle of any textile product, while optimising product longevity and to increase the quality of the recyclates to meet market demands for circularity.

Secondly, education is key. Circularity is a relatively new concept for the complex textile supply chain [180] and is yet to be commercially adopted. The potential downstream effects of certain chemicals and processes, especially in terms of recyclability, are not well known to manufacturers, brands, and, generally, the industry. Engaging all members of the supply chain is crucial to stimulate the development of new business models, the adoption of alternatives with circularity in mind, and new research on potential solutions.

Thirdly, many of the alternative solutions are limited by increased cost. Economic incentives such as tax reductions (as part of the EPR scheme) or penalisation for the use of harmful chemical and methods increase the incentive to move towards alternatives. This is one instance where legislation could have a positive impact on the textile industry.

This paper has focused on recycling polyester at the end of its life, but to become sustainable, waste must be eliminated from the supply chain at all stages of production and use. This includes reducing the over-production, over-consumption, and production of garments and textiles that do not meet adequate standards to be valued by consumers. We must return to designing and developing products to have emotional and physical durability and not simply drive consumption.

## Figures and Tables

**Table 3:** Contamination resulting from use of post-consumer plastics and effects on rPET properties [68].

Contaminant	Effect on rPET Properties
Ink, PVC	Increased reduction in intrinsic viscosity/molar mass
Amosorb, PS, PVC, TiO_2_	Yellowing
PS, PP	Increased haze
PS, PP, EVOH, PVC	Hinders polycondensation during SSP
PLA, EVOH, PVC	Faster crystallisation
PP, PLA	Particle contamination
PVC	Benzene formation
EVOH	Cross-linking

## Data Availability

Data is contained within the article.

## Abbreviations

The following abbreviations have been used in this manuscript:

BD	butane-1,4-diol
BHET	bis(2-hydroxyethyl) terephthalate
BPA	bisphenol-A
CHDM	1,4-cyclohexanedimethanol
DEG	diethylene glycol
DEHP	di(2-ethylhexyl) phthalate
DINP	di-isononyl phthalate
DMT	dimethyl terephthalate
ECHA	European Chemicals Agency
EG	ethylene glycol
EPR	extended producer responsibility
FTOHs	fluorotelomer alcohols
IAS	intentionally added substances
IPA	isophthalic acid
Na_2_CO_3_	sodium carbonate
NaOH	sodium hydroxide
NIAS	non-intentionally added substances
NIR	near-infrared
NP	nonylphenol
OP	octylphenol
PBT	poly(butylene terephthalate)
PD	propane-1,3-diol
PE	polyethylene
PEN	poly(ethylene naphthalate)
PET	poly(ethylene terephthalate)
PFAS	poly- and per-fluoroalkyl substances
PP	polypropylene
PS	polystyrene
PTT	poly(trimethlyene terephthalate)
PVC	poly(vinyl chloride)
RFID	radio frequency identification
rPET	recycled polyethylene terephthalate
ScCO_2_	supercritical CO_2_
SCFP	side-chain-fluorinated polymers
SIPE	5-bis(hydroxyethyl)-isophthalate
TiO_2_	titanium dioxide
TPA	terephthalic acid
